# Estimated energy and nutrient intake in complementary feeding methods in Brazilian infants: randomized clinical trial

**DOI:** 10.1038/s41598-023-50415-7

**Published:** 2024-01-02

**Authors:** Paula Ruffoni Moreira, Muriele Betencourt Silveira, Renata Oliveira Neves, Leandro Meirelles Nunes, Juliana Rombaldi Bernardi

**Affiliations:** 1 Graduate Program in Child and Adolescent Health, Faculty of Medicine, Universidade Federal do Rio Grande do Sul (UFRGS), Rua Ramiro Barcelos 2400, Porto Alegre, RS 90035-003 Brazil; 2 Graduate Program of Food, Nutrition, and Health, Department of Nutrition, Faculty of Medicine, Universidade Federal do Rio Grande do Sul (UFRGS), Porto Alegre, RS Brazil; 3 Hospital de Clínicas de Porto Alegre (HCPA), Porto Alegre, RS Brazil

**Keywords:** Nutrition, Paediatric research

## Abstract

Inadequate nutrient intake during complementary feeding (CF) can affect healthy infant growth and development. A randomized clinical trial was conducted to examine the energy and nutrient intake in Brazilian children randomly assigned to three distinct CF methods. Mother-infant pairs participated in the study, with mothers receiving interventions in one of three CF approaches: (A) strict Parent-Led Weaning (PLW); (B) strict Baby-Led Introduction to Solids (BLISS); and (C) a mixed method. Assessments were made at 5.5 months, nine months, and 12 months of the child's age. Food consumption was measured through 24-h dietary recalls at nine and 12 months, with intake estimates calculated using the Brazilian Food Composition Table. Means or medians of energy and nutrients were compared between groups using ANOVA with Tukey's post hoc test or the Kruskal–Wallis test. A total of 115 infants were evaluated at nine months, and 102 at 12 months. Children in the PLW, BLISS, and mixed method groups exhibited comparable dietary intakes of energy, macronutrients, and micronutrients at both nine and 12 months. Infants following PLW, BLISS, and mixed methods demonstrated similar levels of energy and nutrient intake, underscoring the effectiveness of these strategies in ensuring comparable nutrient intake during the critical phase of CF.

**Trial registration** The trial was registered in the Brazilian Registry of Clinical Trials (ReBEC) with identifier [RBR-229scm U1111-1226-9516], [https://ensaiosclinicos.gov.br/rg/RBR-229scm]. The full data of the first registration was on 24/09/2019.

## Introduction

Proper and healthy eating habits during the early years of life, particularly within the crucial first 1000 days, are essential for fostering a child's optimal growth and development^1^. Exclusive breastfeeding up to the sixth month, followed by the introduction of nutritionally adequate complementary foods aligned with family traditions, forms the foundation for a nutritious diet in children under 2 years of age. Traditionally, it has been recommended to introduce complementary foods (CF) mashed with a fork and offered with a spoon by an adult to child^2^. This recommendation is based on the perception that infants may be immature in their ability to chew and swallow whole foods. However, around 6 months of age, most children reach a stage of development with physiological and neurological maturity, along with a reduced tongue protrusion reflex, allowing them to consume solid foods with complex textures, including soft whole foods^3^.

In light of the physiological and neurological maturity of 6-month-old infants, alternative methods for introducing CF have emerged, suggesting that a gradual transition in textures may not be necessary^4^. Among these methods, two prominent approaches have gained widespread popularity: Baby-Led Weaning (BLW) and Baby-Led Introduction to Solids (BLISS). BLW, introduced in 2008 by author Gill Rapley in her book "Baby-led Weaning: Helping Your Baby to Love Good Food"^5^, advocates for offering whole foods and encouraging self-feeding right from the start of the CF period. Similarly, BLISS, introduced in 2015 by a group of New Zealand researchers^6^, emphasizes self-feeding and encourages the consumption of whole foods, discouraging the use of purees. Both methods promote the child's autonomy during meals, offering the same meals as the parents and encouraging communal culinary experiences within the family^7^.

Research evaluating the safety of child-led feeding methods, such as BLW and BLISS, has shown no increased risk of choking incidents^8^, insufficient iron intake^9^, low plasma ferritin levels^9^, or poor infant growth^10^. Conversely, infants following a Parent-Led Weaning (PLW) approach exhibited higher sodium and sugar intake^11^, more total and saturated fat^11^, and a lower intake of iron and vitamin B12^7,12,13^.

Despite the growing popularity of BLW and BLISS in Brazil, there is limited evidence regarding the safety of these methods in Brazilian children. Additionally, healthcare professionals are concerned about whether children fed with greater autonomy achieve sufficient nutrient intake^14^. This study aims to address these concerns by analyzing the estimated energy and nutrient intake in infants allocated to three different CF approaches: PLW, BLISS, and a mixed approach. By comparing the nutrient intake of infants following these methods, we can gain valuable insights into the effectiveness of these strategies in ensuring adequate nutrition during the critical phase of CF.

## Methods

A randomized clinical trial comparing complementary feeding methods in mother-infant pairs: (A) strict PLW, which is the control arm; (B) strict BLISS; and (C) mixed method: a combination of PLW and BLISS created especially for this study. Additional information about the randomized trial can be found in the published study protocols^15,16^.

Participants were recruited online between 2019 and 2020 through social media platforms, the university's online community, a university hospital's online network, healthcare professionals' social media groups, and mother-focused social media groups. The invitation to take part in the study included the researcher’s phone numbers and email addresses, allowing interested mothers to get in touch. Upon contacting the researchers, mothers were queried about the inclusion criteria and given instructions regarding the research protocols.

Inclusion criteria specified mothers residing in Porto Alegre, Rio Grande do Sul, Brazil, or nearby cities, with healthy singleton infants born at term, weighing ≥ 2500*g*, having internet access, and not having initiated the complementary feeding process. Children lacking data on food consumption at 9 and 12 months were excluded from the study.

Upon signing the consent form, participants were sequentially numbered and had their names entered into a randomization list divided into three blocks with equal numbers, generated beforehand using http://www.randomization.com by research staff blinded to the process. All families were instructed to delay complementary feeding until the sixth month. Only at the time of the first intervention were mothers informed of their group allocation. Data assessment was conducted by a different researcher, not involved in the intervention, who was blinded to the allocation group, in a separate room.

All participants received one of the three interventions. Initially, when infants reached 5.5 months, a dietary workshop was conducted in a private nutrition office equipped with an experimental kitchen. Nutritionists guided parents on appropriate complementary feeding methods according to their assigned group. The workshop, lasting approximately 45 min, was conducted in groups of eight mothers, allowing participants and nutritionists to prepare example meals together in real-time.

During the same visit, speech therapists provided advice on choking prevention, utilizing videos demonstrating choking and gagging incidents for approximately 15 min. All advice was standardized; each group received specific printed material addressing topics covered by the professional, and participants were provided with copies to take home. All groups were also given information on continued breastfeeding and healthy feeding. The primary differences between the intervention groups pertained to the consistency of foods and the level of adult participation during each meal.

The intervention groups were as follows.

A. Strict Parent-Led Weaning group: Mothers in this group, in addition to receiving standard information, were encouraged to spoon-feed pureed foods to their infants, following the traditional feeding method^2^.
B. Strict BLISS group: Participants in this group, along with standard information, were taught to prepare meals shaped into sticks, allowing children to self-feed without adult interference^6^.
C. Mixed group: This group was instructed to combine the above-mentioned methods according to the child’s preferences.

During the second interview, conducted during a home visit when infants were nine months old, a 24-h dietary recall was administered. Individualized advice or support for the complementary feeding process was provided as needed, tailored to the approach assigned to each mother-child pair. During the COVID-19 pandemic, the 24-h dietary recall was conducted over the phone, and online support for complementary feeding was provided. The interviews and 24-h dietary recalls at 12 months were conducted at the Hospital de Clínicas de Porto Alegre (HCPA) in the Centro de Pesquisa Clínica (CPC). During the pandemic, the 24-h dietary recall was also conducted over the phone, and online support for complementary feeding was provided.

The 24-h dietary recalls were administered by trained researchers, referring to the preceding day. They aimed to estimate the quantity, type, brands, time of day, and cooking methods (including descriptions of any recipes used and quantities of raw ingredients) for all foods and drinks consumed by the infants. If the mother considered the previous day atypical, she was instructed to replace it with a day of the week more representative of the family's typical eating routine.

All diet records were entered into the dietary analysis software Dietbox^®^ (using the Brazilian Food Composition Table and Food Composition Table: support for nutritional decision^17^) to analyze energy (Kcal/day), carbohydrate (g/day), total protein (g/day), total lipids (g/day), saturated fat (g/day), monounsaturated fat (g/day), polyunsaturated fat (g/day), cholesterol (mg/day), total fibers (g/day), calcium (mg/day), sodium (mg/day), vitamin C (mg/day), vitamin B9 (mcg/day), iron (mg/day), zinc (mg/day), and total sugar (g/day).

Sociodemographic data for the families were collected through an online questionnaire completed after signing the consent form at the start of the study. The questionnaire included maternal age (years), maternal education (university or high/basic school), total family income (Brazilian real), maternal parity (first child or not), maternal race/ethnicity (white or non-white, including brown, black, yellow, or indigenous), maternal marital status (with or without a partner), sex of the infant (female or male), and infant birth weight (kilograms).

At nine and 12 months, mothers answered questions about the duration of exclusive breastfeeding (EBF) and the type of feeding (breastfeeding, infant formula, or a combination of both). EBF was defined as when the child received no liquid or solid other than human milk—no water, oral rehydration solution, or drops/syrups of vitamins, minerals, or medications. Any breastfeeding practice was defined as receiving any amount of human milk by bottle, cup, or breast, regardless of any other food offerings^18^. Additionally, mothers provided information on the provision of meat and eggs (iron-rich foods) during lunch and dinner at nine and 12 months, as well as iron supplement consumption at these ages.

The sample size for the main outcome (body mass index—BMI—for age) was calculated using WinPepi^®^ software version 11.65, based on previously published studies involving similar subjects^6,7^. With a unit standard deviation of 1, a statistical power of 80%, and a significance level of 5% to detect a difference in BMI of 0.8 kg/m^2^, the sample size for a difference of half a standard deviation consisted of 48 mother-infant pairs for each of the three intervention groups, totaling 144 mother-infant pairs.

The database was created using Statistical Package for the Social Sciences (SPSS)^®^ software version 21.0, with double data entry and subsequent validation. The analysis followed the intention-to-treat principle. The normality of quantitative variables was assessed using the Kolmogorov-Smirnov test. Quantitative variables were described as mean and standard deviation (± SD) or median and interquartile range [P25–P75], while categorical variables were presented as absolute (n) and relative (%) frequencies. Means or medians of energy, carbohydrate, total protein, total lipids, saturated fat, monounsaturated fat, polyunsaturated fat, cholesterol, total fibers, calcium, sodium, vitamin C, vitamin B9, iron, zinc, and total sugar were compared between groups using ANOVA with Tukey's post hoc test or Kruskal–Wallis test.

This study was approved by the Research Ethics Committee of Hospital de Clínicas de Porto Alegre (approval number: 2019-0230, CAAE: 1537018500005327) and was conducted in accordance with relevant guidelines and regulations, following Resolution number 466 of December 12, 2012, of the National Health Council of Brazil. Written informed consent to participate in this study was obtained from the legal guardian(s) of the participant.

The trial was registered in the Brazilian Registry of Clinical Trials (ReBEC) under the identifier RBR-229scm U1111-1226-9516 (https://ensaiosclinicos.gov.br/rg/RBR-229scm). The initial registration data were recorded on September 24, 2019.

## Results

Initially, a total of 207 mothers expressed interest in participating in the research between 2019 and 2020, out of which 12 did not meet the inclusion criteria. As a result, we identified 195 eligible mother-infant pairs, and subsequently, 145 mothers were randomized into three groups: n = 47 for the PLW Method, n = 49 for the BLISS method, and n = 49 for the mixed method.

**Figure 1:**
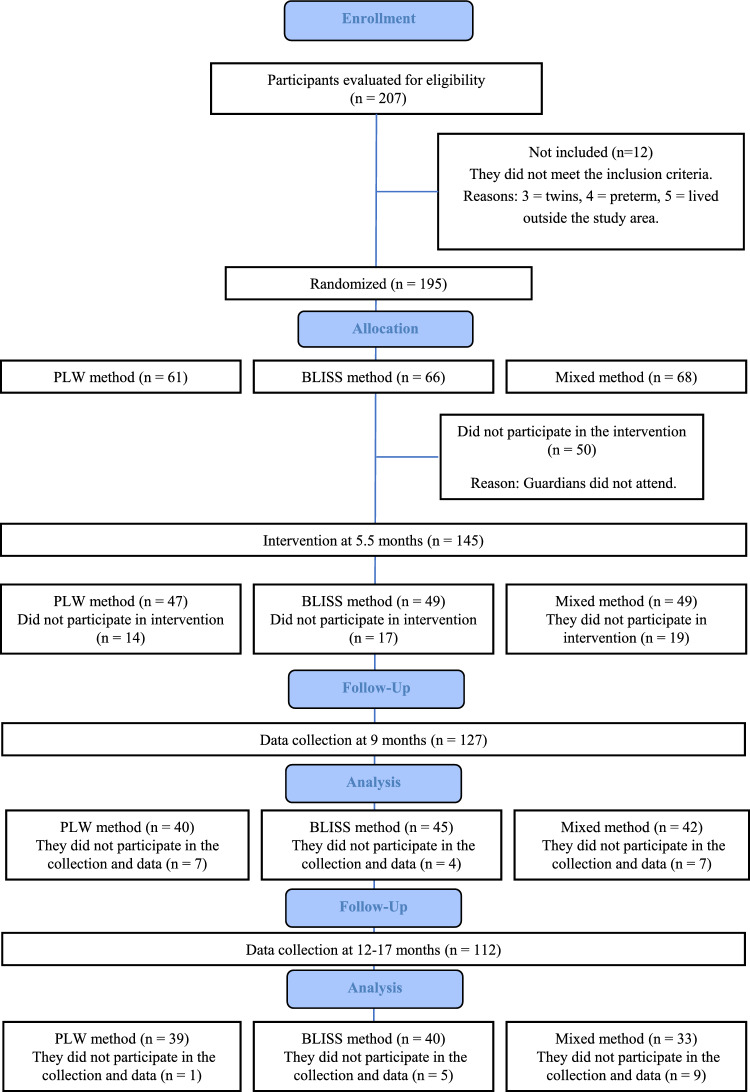
Study flow diagram. *BLISS:* Baby-Led Introduction to SolidS, *PLW:* Parent-Led Weaning.

At 9 months, a total of 115 children were assessed, with 35 in the PLW group, 42 in the BLISS group, and 38 in the mixed group. At 12 months, 102 children were assessed, with 40 in the PLW group, 38 in the BLISS group, and 24 in the mixed group (Figure 1). Table 1 presents the sociodemographic characteristics of the sample. In the overall sample, the median maternal age was 38 [33–41] years, and the median family income was 6 [4–10] thousand Brazilian reais. Moreover, 85.8% (n = 109) of the mothers had a university education, and 85.8% (n = 109) resided with a partner. Regarding the type of dairy feeding at 9 months, 55.9% (n = 71) of the children were breastfed, 15.7% (n = 20) were solely on formula milk, and 27.6% (n = 35) were on mixed feeding, involving breast milk, formula milk, and cow's milk. At 12 months, 51.6% (n = 65) of the children were breastfed, 20.5% (n = 26) were on formula milk alone, and 27.9% (n = 35) were on mixed feeding with breast milk, formula milk, and cow's milk.

**Table 1:** Characteristics of PLW, BLISS, and Mixed study participants who provided estimated intake data, at 9 and 12 months of age.

Variables	Methods of complementary feeding		
	PLW (*n* = 40)	BLISS (*n* = 45)	Mixed (n = 42)
Family variables	n (%)/median [P25–P75]		
Maternal age (years) (n = 126)	38 [31–41]	38 [36–42]	36 [32–39]
Maternal education (n = 127)			
University	32 (80.0)	40 (88.9)	37 (88.1)
High/basic school	8 (20.0)	5 (11.1)	5 (11.9)
Family total income (n = 126)	5 [3–10]	8 [4–14]	5 [3–10]
Maternal parity (n = 127)			
First child	30 (75.0)	34 (75.6)	38 (90.5)
Maternal race/ethnicity (n = 126)			
White	32 (82.1)	39 (86.7)	37 (88.1)
Maternal marital status (n = 127)			
With partner	32 (80.0)	41 (91.9)	36 (85.7)
Sex of the infant (n = 127)			
Female	21 (52.5)	24 (53.3)	19 (45.2)
Infant birth weight (kg) (n = 127)	3.2 [2.9—3.8]	3.3 [3.0—3.4]	3.2 [2.9—3.6]
Diet infant variables	n (%)/median [P25-P75]		
Exclusive breastfeeding (days) (n = 125)	180 [97—180]	180 [150—180]	180 [150—180]
Started eating (days) (n = 121)	180 [171—180]	180 [180—180]	180 [180—180]
Type of feeding at 9 months (n = 127)			
Breastfeeding	19 (47.5)	27 (60.0)	25 (59.5)
Infant formula	4 (10.0)	9 (20.0)	7 (16.7)
Breastfeeding and Infant formula	16 (40.0)	8 (17.8)	10 (23.8)
Type of feeding at 12 months (n = 126)			
Breastfeeding	17 (42.5)	27 (60.0)	21 (51.2)
Infant formula	6 (15.0)	10 (22.2)	10 (24.4)
Breastfeeding and Infant formula	13 (32.5)	6 (13.3)	6 (14.6)

*BLISS:* Baby-Led Introduction to SolidS, *PLW:* Parent-Led Weaning, Percentile.

The median duration of exclusive breastfeeding (EBF) was 180 days in all groups, with interquartile ranges ranging from 97 to 180 days in the PLW group, 150–180 days in the BLISS group, and 150–180 days in the mixed group. There were no statistically significant differences found between the groups (p = 0.384). Regarding the introduction of infant food solids, the median was 180 [171–180] days in the PLW group, 180 [180–180] days in the BLISS group, and 180 [180–180] days in the mixed group. Similarly, no differences were observed between the methods (p = 0.862). Additionally, there were no differences in the type of infant milk consumed at nine or 12 months (p = 0.240 and p = 0.312, respectively).

Concerning infant energy consumption (kcal/day) at 9 months, the overall mean was 426.5 kcal (± 214.2), with 427.0 kcal (± 204.5) in the PLW group, 406.7 kcal (± 208.3) in the BLISS group, and 447.0 kcal (± 232.0) in the mixed group. No significant difference was observed between the methods of food introduction (p = 0.684). For macronutrient consumption, the estimated average carbohydrate intake for the day was 64.1 g (± 31.8), with no significant difference between the methods (p = 0.958). The median total protein consumption for the day was 18.4 g [12.6–30.0], and no differences were observed between the methods (p = 0.431). Additionally, regarding lipids, the median estimated daily consumption was 8.3 g [4.6–15.4], with no differences between the methods of food introduction (p = 0.371). Likewise, no differences were observed in micronutrient consumption among the groups (p > 0.05) (Table 2).

**Table 2:** Estimated nutrient intake of infants submitted to three complementary feeding methods, at 9 and 12 months.

Methods of complementary feeding				
	PLW	BLISS	Mixed	*p*-value
Estimated nutrient intake at 9 months (n = 127)				
Estimated nutrient intake at 12 months (*n* = 112)				

Variables	Mean (± SD)/Median [P25–P75]			
Energy (kcal/day)				
9 months	427.0 (204.5)	406.7 (208.3)	447.0 (232.0)	0.684
12 months	637.8 (228.8)	610.4 (252.4)	605.3 (252.7)	0*.*812
Carbohydrate (g/day)				
9 months	65.3 (29.5)	63.4 (32.3)	63.6 (34.0)	0.958
12 months	93.8 [65.7–115.4]	81.5 [62.3–112.0]	87.2 [58.3–115.8]	0.761
Total protein (g/day)				
9 months	17.9 [11.7–28.5]	21.8 [13.3–32.8]	21.8 [13.3–32.8]	0.431
12 months	31.6 [19.9–40.2]	24.9 [19.9–36.5]	27.4 [18.2–39.9]	0.572
Total lipids (g/day)				
9 months	9.5 [4.6–15.7]	8.4 [5.7–16.2]	8.4 [5.7–16.2]	0.371
12 months	16.8 [10.1–23.5]	12.7 [6.9–22.7]	10.2 [6.9–22.9]	0.316
Saturated fat (g/day)				
9 months	2.4 [1.2–4.4]	2.0 [0.6–3.4]	2.7 [1.2–4.4]	0.165
12 months	5.0 [2.6–7.0]	4.4 [2.7–6.1]	5.0 [2.6–7.0]	0.485
Monounsaturated fat (g/day)				
9 months	3.1 [1.2–5.5]	3.0 [1.8–6.0]	3.0 [1.7–6.0]	0.278
12 months	4.0 [2.7–6.3]	3.5 [1.6–6.1]	3.2 [2.0–7.2]	0.450
Polyunsaturated fat (g/day)				
9 months	1.6 [0.7–2.8]	1.4 [0.9–2.8]	1.4 [0.1–2.8]	0.081
12 months	2.2 [1.2–2.9]	1.9 [1.2–3.9]	1.8 [1.0–3.3]	0.892
Trans fat (g/day)				
9 months	0 [0–0.1]	0 [0–0]	0 [0–0]	0.173
12 months	2.1 [0–14.7]	1.2 [0–15.1]	1.9 [0–14.3]	0.797
Cholesterol (mg/day)				
9 months	46.2 [17.6–128.7]	69.6 [29.3–166.3]	69.6 [29.3–166.3]	0.674
12 months	115.7 [44.4–242.4]	117.6 [43.9–248.6]	101.6 [37.6–233.7]	0.717
Total fibers (g/day)				
9 months	11.3 (5.9)	11.20 (6.2)	11.4 (6.3)	0.985
12 months	10.5 [7.0–17.7]	12.3 [8.8–18.2]	11.7 [8.0–17.1]	0.762
Calcium (mg/day)				
9 months	81.8 [56.1–152.8]	92.8 [53.1–157.8]	103.8 [57.7–139.3]	0.989
12 months	138.9 [82.8–201.6]	131.4 [131.4–190.7]	113.9 [82.6–187.0]	0.596
Sodium (mg/day)				
9 months	140.0 [55.6–239.6]	130.0 [63.9–369.3]	130.0 [63.9–369.3]	0.583
12 months	304.3 [183.8–500.0]	301.6 [163.3–600.1]	392.2 [191.2–628.2]	0.695
Vitamin C (mg/day)				
9 months	67.3 [24.1–111.8]	59.1 [32.5–113.3]	59.1 [32.5–113.3]	0.645
12 months	61.7 [31.9–95.5]	65.6 [39.0–129.2]	59.5 [30.6–81.1]	0.639
Vitamin B9 (mcg/day)				
9 months	11.4 [0–38.6]	16.3 [0–93.9]	16.3 [0–93.9]	0.273
12 months	32.5 [5.8–87.8]	32.4 [3.7–80.4]	28.5 [2.1–99.6]	0.876
Iron (mg/day)	3.3 [1.9–4.4]	3.1 [1.8–5.3]	3.1 [1.8–5.3]	0.923
9 months	3.3 [1.9–4.4]	3.1 [1.8–5.3]	3.1 [1.8–5.3]	0.923
12 months	4.5 [2.9–6.2]	5.05 [3.1–6.2]	4.5 [2.9–6.6]	0.972
Zinc (mg/day)				
9 months	2.2 [1.5–4.1]	2.5 [1.6–4.4]	2.5 [1.6–4.4]	0.667
12 months	3.5 [2.4–5.0]	3.1 [2.4–5.3]	3.9 [1.9–6.0]	0.745
Total sugar (g/day)				
9 months	0.17 [0–4.8]	0.2 [0–9.9]	0.2 [0–9.9]	0.458
12 months	2.1 [0–14.7]	1.2 [0–15.1]	1.9 [0–14.3]	0.797

*BLISS:* Baby-Led Introduction to SolidS, *PLW:* Parent-Led Weaning, *SD:* Standard deviation, Percentile.

At 12 months, the overall mean energy consumption (kcal/day) was 610.2 kcal (± 247.1), with 637.8 kcal (± 228.8) in the PLW method, 610.4 kcal (± 252.4) in the BLISS method, and 605.3 kcal (± 252.7) in the mixed method. No statistically significant difference was observed in intake estimation among the complementary feeding methods (p = 0.812). Regarding macronutrient consumption, the overall median carbohydrate intake for the day was 87.9 g [62.3–115.6], and no differences were observed between the methods (p = 0.761). The median total protein intake for the day was 26.8 g [18.5–37.1] in the total sample, with no differences between the methods (p = 0.572). Moreover, for total lipids, the overall median estimated daily consumption was 12.8 g [7.5–22.2], with no observed differences between the methods (p = 0.316). Similarly, there were no differences in micronutrient consumption between the groups (p > 0.05) (Table 2).

Regarding lunch, meat and eggs were included in 92% (n = 37) of the PLW group's children's diet, 92% (n = 37) in the BLISS group, and 97% (n = 35) in the mixed group at 9 months (p = 0.321). For dinner, 80% (n = 32) of children in the PLW group were served meat and eggs, compared to 76% (n = 34) in the BLISS group and 71% (n = 30) in the mixed group at 9 months (p = 0.545). At 12 months, meat and eggs were included in the lunch for 95% (n = 38) of children following the PLW method, 96% (n = 43) in the BLISS method, and 90% (n = 38) in the mixed method (p = 0.661). Regarding dinner, meat and eggs were included in the meals of 85% (n = 34) of children in the PLW method, 89% (n = 40) in the BLISS method, and 81% (n = 34) in the mixed method (p = 0.612). The consumption of iron and zinc sources was similar across the CF approaches. Iron supplements were consumed by 85% (n = 34) of infants in the PLW method, 75% (n = 34) in the BLISS method, and 75% (n = 35) in the mixed method at 9 months (p = 0.596). At 12 months, 72% (n = 29) of infants in the PLW method received iron supplements, 73% (n = 33) in the BLISS method, and 73% (n = 30) in the mixed method, with no differences between groups (p = 0.996).

## Discussion

This study investigated the estimated intake of energy, macronutrients, and micronutrients in children whose mothers received nutritional guidance for complementary feeding (CF), following three distinct approaches: Parent-Led Weaning (PLW), Baby-Led Introduction of SolidS (BLISS), and a mixed method, determined through 24-h dietary recalls. Importantly, no statistically significant differences were observed in the estimated intake of energy, macronutrients, and micronutrients from solid foods at 9 and 12 months of age.

Nutrient intake during childhood plays a crucial role in cognitive and motor development and is closely linked to the risk of developing obesity^19,20^. The increasing popularity of child-led CF methods, especially BLISS, has raised concerns among healthcare professionals, parents, and caregivers about potential nutritional deficiencies^14^. However, this study demonstrates that the examined approaches resulted in similar estimated quantities of energy, macronutrients, and micronutrients.

The dietary intake of children following different CF approaches has been analyzed in other randomized clinical trials. A study in New Zealand that introduced the BLISS method analyzed the feeding patterns of 206 infants, with 101 in the control group (PLW) and 105 in the BLISS group. At 7 months, infants in the BLISS group consumed higher amounts of lipids (in grams) and sodium (in mg) compared to infants in the control group. However, this difference was not observed at 12 months^6^. Additionally, at 12 months, BLISS infants consumed a lower percentage of calories from saturated fat. Similar to the present study, no differences were found in energy and macronutrients, except for total lipids at 7 months^11^. Another randomized controlled trial in Turkey evaluated the iron intake of 280 infants, with 146 fed with a spoon (PLW) and 156 following the BLW method^21^. No significant differences were found between the two groups of infants at 12 months. Energy and macronutrient intake were not assessed. The results of this study support the findings analyzed. However, it is essential to note that both studies analyzed solid and dairy foods together; therefore, the difference in intake cannot be exclusively attributed to the methods of CF.

Studies with methodological weaknesses have also examined nutrient intake in different complementary feeding approaches. A cross-sectional study showed that 36 children fed by BLW consumed less vitamin B12 than infants fed by the traditional spoon-feeding method. Other differences were observed in milk intake, but regarding solid foods, only vitamin B12 showed significant differences^13^. Another cross-sectional study demonstrated that children following the BLW approach consumed a lower percentage of energy from proteins, fibers, iron, zinc, vitamin C, vitamin B12, and calcium compared to children fed by the traditional spoon-feeding method^22^. Although these results seem inconsistent with the findings of the present study, it is essential to recognize significant differences in the methodology used to assess intake estimates. Furthermore, in both studies, parents spontaneously embraced the BLW method, whereas in the present study, parents were guided to adopt the BLISS approach after receiving instructions.

Concerns about iron intake are particularly relevant for children following self-feeding approaches, as these methods encourage the consumption of natural foods such as fruits and vegetables that contain small amounts of iron^9^. However, in our study, after intervention guiding the offering of iron-rich foods in BLISS and mixed methods, no lower iron consumption was observed compared to spoon-feeding. Other studies that included intervention also found similar iron intake quantities^9,21^. Therefore, it seems reasonable to state that child-led approaches, when guided by a healthcare professional, provide similar amounts to traditional spoon-feeding methods of this essential nutrient for proper child development. The same might not be valid for families who adopt this method spontaneously, as Pearce and Langley-Evans' results demonstrated lower iron intake in BLW-fed children^13^. The estimated iron intake from solid foods in complementary feeding shown in this study does not meet the needs of this nutrient for this age group. As complementary feeding is not the primary source of nutrients in the first year of life, this recommendation is likely to be met through breastfeeding and/or formula^2^. Moreover, in Brazil, iron supplementation is recommended for all children between 6 and 24 months as a preventive measure^23^. A substantial difference in this present study compared to other randomized controlled trials is that we did not recommend the consumption of iron-fortified cereals. In Brazil, these foods are not recommended for children under 2 years old^2^.

However, this study has some limitations in the collection of food consumption data. A 24-h recall was used per period, instead of three as in other studies, and families did not weigh food but reported the food consumed in homemade measures, which may overestimate or underestimate consumption. Additionally, our recruitment through the Internet might have led to a higher number of mothers with better socioeconomic status and more interest in complementary feeding participating in the research, limiting the external validity of these data. Because the study was analyzed by intention to treat, which is recommended in clinical trials, adherence to the methods was not considered in this study. Another study limitation is the limited adherence to the proposed methods in randomization. Our results showed that approximately 30% of the children continued to follow the recommended method^24,25^. In general, families showed greater adherence to the mixed method, which combined both techniques (PLW and BLISS).

Notwithstanding these limitations, this study yields valuable insights into the estimated nutrient intake in child-led and parent-led complementary feeding methods among Brazilian children. In summary, the findings of this research highlight that complementary feeding approaches, including PLW, BLISS, and mixed methods, result in comparable estimated intake levels of energy, macronutrients, and micronutrients at both nine and 12 months of age. While this study contributes significant data to the field, we recommend further observational studies and randomized clinical trials, with a particular focus on the methodology used to assess nutrient intake. When guided by a nutritionist, all tested complementary feeding methods offer similar quantities of energy, macronutrients, and micronutrients for Brazilian children in their first year of life.

## Data Availability

The datasets generated and/or analyzed during the current study are not publicly available due to longitudinal data collection being ongoing but are available from the corresponding author upon reasonable request.
